# Oncolytic viruses as therapeutic cancer vaccines

**DOI:** 10.1186/1476-4598-12-103

**Published:** 2013-09-11

**Authors:** David L Bartlett, Zuqiang Liu, Magesh Sathaiah, Roshni Ravindranathan, Zongbi Guo, Yukai He, Zong Sheng Guo

**Affiliations:** 1 University of Pittsburgh Cancer Institute and Department of Surgery, University of Pittsburgh, Pittsburgh, PA 15213, USA; 2 Fujian Huitian Biopharmaceuticals, Ltd., Sanming, Fujian 365001, China; 3 Immunology/Immunotherapy Program, Georgia Regents University Cancer Center, Augusta, GA 30912, USA

**Keywords:** Oncolysis, Immunogenic cell death, Autophagy, Antigen, Cross-presentation, Antitumor immunity, Immunotherapy, Cancer vaccine

## Abstract

Oncolytic viruses (OVs) are tumor-selective, multi-mechanistic antitumor agents. They kill infected cancer and associated endothelial cells via direct oncolysis, and uninfected cells via tumor vasculature targeting and bystander effect. Multimodal immunogenic cell death (ICD) together with autophagy often induced by OVs not only presents potent danger signals to dendritic cells but also efficiently cross-present tumor-associated antigens from cancer cells to dendritic cells to T cells to induce adaptive antitumor immunity. With this favorable immune backdrop, genetic engineering of OVs and rational combinations further potentiate OVs as cancer vaccines. OVs armed with GM-CSF (such as T-VEC and Pexa-Vec) or other immunostimulatory genes, induce potent anti-tumor immunity in both animal models and human patients. Combination with other immunotherapy regimens improve overall therapeutic efficacy. Coadministration with a HDAC inhibitor inhibits innate immunity transiently to promote infection and spread of OVs, and significantly enhances anti-tumor immunity and improves the therapeutic index. Local administration or OV mediated-expression of ligands for Toll-like receptors can rescue the function of tumor-infiltrating CD8^+^ T cells inhibited by the immunosuppressive tumor microenvironment and thus enhances the antitumor effect. Combination with cyclophosphamide further induces ICD, depletes Treg, and thus potentiates antitumor immunity. In summary, OVs properly armed or in rational combinations are potent therapeutic cancer vaccines.

## Introduction

In the last few years, there is mounting evidence that OVs are effective in treating cancer in both preclinical models and clinical trials with human patients [1-3]. The anticancer activities of OVs are derived from multimodal cancer killing mechanisms. The first is the direct oncolysis of cancer cells by the virus, which is, in most cases a mixture of apoptosis, necrosis, pyroptosis and autophagic cell death, often with one as predominant for a particular OV. The second is apoptotic and necrotic death of uninfected cells induced by anti-angiogenesis and anti-vasculature of the OVs as shown in animals and humans [4-6]. The last is cytotoxicity to cancer and stromal cells by activated innate and tumor-specific immune cells [7-9]. The antitumor immunity helps eliminate the uninfected cancer cells in primary and metastatic nodules, and enforce micrometastases in dormant state.

Cancer vaccines are designed to boost the body’s immune system to protect itself from carcinogenesis and progression of cancer. The Food and Drug Administration of the USA has approved both prophylactic and therapeutic vaccines for cancer in the last few years. The prophylactic vaccines are against the hepatitis B virus, which can cause liver cancer, and against human papillomavirus types 16 and 18, which are responsible for about 70 percent of cervical cancer cases. These anti-viral vaccines are highly effective in curbing virus infections and onset of cancer. In contrast, therapeutic cancer vaccines are difficult to develop and much less effective. As a benchmark, Provenge, a cancer vaccine designed to treat advanced prostate cancer, demonstrated an increase in survival and thus gained approval from the FDA for use in the treatment of advanced prostate cancer patients in 2010 [10]. The approval of Provenge has stimulated interest in development of other therapeutic cancer vaccines.

OVs provide a number of potential advantages as cancer vaccines over conventional therapies. First, OVs are tumor-selective, thus *in situ* cancer vaccines, providing higher cancer specificity and better safety margin. Second, immunogenic/inflammatory types of cell death, including recently characterized “immunogenic cell death” (ICD) of cancer and stromal cells induced by OVs provides a natural repertoire of tumor-associated antigens (TAAs) in conjunction with danger signals [damage-associated molecular pattern (DAMP) and OV-derived pathogen-associated molecular pattern (PAMP) molecules, and inflammatory cytokines] [11-13], to elicit anti-tumor immunity. However, just like other immunotherapeutic regimens, a number of challenges remain for OVs-mediated cancer vaccines. For example, the relative inefficiency of delivering OVs to tumor nodules, selective viral replication inside tumor nodules and spread to distant micrometastases limits its overall efficacy. In order to make up this deficiency, it often requires combinations with conventional treatments for cytoreduction to decrease the tumor burden. Most TAAs are self-antigens and thus weakly immunogenic. In addition, a highly immunosuppressive tumor microenvironment (TME) often suppresses the activities of tumor-infiltrated lymphocytes (TILs) generated spontaneously, by adoptive cell transfer or by active immunization such as cancer vaccines. Therefore, the balance between tumor growth and the status of the TME, versus the magnitude and avidity of antitumor immune response elicited by a therapeutic vaccine in addition to oncolytic potency by an OV ultimately determines the therapeutic efficacy by this approach [9,14-17].

In this review, we briefly introduce oncolytic virotherapy and cancer immunotherapy, then focus on the rationales and strategies of utilizing replicating OVs as therapeutic cancer vaccines, and combination strategies that have led to potent antitumor immunity in preclinical models and demonstration of the effectiveness of two OVs in clinical trials.

### OVs and cancer immunotherapy

OVs possess the ability to selectively infect and replicate in cancer and associated endothelial cells and kill these cells in cancerous tissues while leaving normal tissues unharmed [1,3]. Many naturally occurring OVs have a preferential tropism for tumor and/or associated endothelial cells. Others are genetically engineered to change their cellular or organ tropism to cancer. The mechanisms of tumor targeting by OVs, which include selectivity to cancer cells and/or associated endothelial cells with altered signaling pathways of RB/E2F/p16, p53, PKR, EGFR, Ras, Wnt, anti-apoptosis, hypoxia conditions, or defects in IFN and other cellular innate immune signaling pathways have been reviewed [1,3,18]. The altered signaling pathways foster favorable cellular environments for specific OVs to replicate sufficiently in cancer cells and/or associated endothelial cells, leading to direct oncolysis of the infected cells.

Viruses often display specificity for a cell type, tissue or species, collectively known as viral tropism. Cytokines, particularly interferons and tumor necrosis factors, play key roles in dictating the viral tropism [19,20]. Complement system seems to play certain roles, as shown for Newcastle disease virus [21]. OVs also displayed species specificity even though they broaden their tropism to cancer cells from non-permissive species to various degrees. Myxoma virus, a poxvirus previously considered rabbit specific, can replicate productively in a variety of human tumor cells [22]. Bovine herpes virus type 1 is a species-specific virus that fails to induce cytopathic effects in human normal cells, yet is capable of infecting and killing a variety of immortalized and transformed human cell types [23]. However, human Ad can infect murine cancer cells yet the production of infectious virus progeny is often limited. One reason is the failure of translation of viral mRNAs and this could be overcome partially by expression of L4-100 K *in trans*[24]. It is important to note that OVs show aberrant, non-productive infection in non-native hosts such as mouse cells. In this case, the resulting mode of cell death can considerably differ from oncolysis in human cancer cells. As we will discuss later, the mode of cell death dictates to a large degree the subsequent antitumor immunity. Consequently, the antitumor immunity determined by studies in immunocompetent animal models with syngeneic tumors might not be relevant to the situation in human cancer patients.

OVs mediate multimodal killing of cancer and stromal cells ranging from direct virus-mediated cytotoxicity [25-28], cell death due to anti-angiogenesis and vasculature targeting by OVs, to cytotoxic immune effector-induced cytotoxicity. The types of cell death, as classified by morphological and ultrastructural changes during cell death, are apoptosis, necrosis, and autophagic cell death. With the exception of apoptosis, all other types of cell death have been considered to be inflammatory and immunogenic. However, recent studies by investigators working on chemotherapy and radiation for cancer therapy have led to new concepts, that apoptotic cell death can be divided into “immunogenic cell death” (ICD) and “non-immunogenic cell death” (NICD) [29-31]. Based on this new classification, apoptotic cell death caused by some OVs are ICD. Together, immunogenic apoptosis, necrosis, autophagic cell death and pyroptosis of cancer and associated endothelial cells caused by OVs release and present danger signals (DAMPs and PAMPs as signal 0) and TAAs (as signal 1) to dendritic cells (DCs) for antitumor and antiviral immune responses.

Immunotherapy has been a bright spot in the field of novel therapeutics for cancer in the last few years [32-34]. Tumor cells and associated stromal cells such as endothelial cells express a wide variety of proteins that can function as antigens including mutated proteins, fusion proteins, developmentally and tissue-restricted proteins, as well as tumor-selectively over-expressed proteins, termed as TAAs [17]. These TAAs are direct targets for most immunotherapeutic regimens, either active immunization or adoptive transfer of activated immune cells [14,33,34]. The TME, in which cancer cells, stromal cells and infiltrated immune cells, as well as soluble molecules interact with each other and dictate its properties, is immune tolerangenic or more likely immunosuppressive [35]. However, the TME and associated signaling pathways can be manipulated to activate antitumor immunity in a therapeutic regimen. Thus, a number of immunotherapeutic strategies are aimed to disrupt the immune-regulatory circuits that are critical for maintaining tumor tolerance, such as CTLA-4 and PD-1, and augment protective antitumor immunity [15,35-37].

### OV-induced ICD and autophagy elicit antitumor immune responses

The cell death can be classified according to morphologic and ultrastructural changes of dying cells, into apoptosis, necrosis, autophagic cell death, pyroptosis and a few other types of death [38,39]. As stated, necrosis, pyroptosis and autophagic cell death are proinflammatory and immunogenic. Necrosis release DAMPs from dying cells. Autophagic cell death also releases many DAMPs. Pyroptosis, triggered by pathogens [40], is highly inflammatory. The only exception is apoptosis. Apoptotic cell death was considered to be non-immunogenic and non-inflammatory by nature (Table 1). However, recent studies suggest that, under certain circumstances, apoptosis can be ICD [29,30,41,42]. ICD involves changes in the composition of the cell surface as well as the release of soluble mediators, occurring in a defined temporal sequence. At the early phase of immunogenic apoptosis, surface-exposed calreticulin (ecto-CRT) and secreted ATP are crucial DAMPs [43]. While calreticulin (CRT) exposure on the cell surface prior to apoptosis dictates the immunogenicity of cancer cell death [29,30,41,42], ERP57 is a key protein that controls immunogenicity by controlling CRT exposure [44,45]. In response to ICD inducers, activation of endoplasmic reticulum (ER) stress is indispensable to confer the immunogenic character of cancer cell death, because ER stress can coordinate the danger signaling pathways responsible for the trafficking of vital DAMPs and subsequent anti-cancer immune responses. Other pathways such as autophagy (discussed later) have the ability to influence danger signaling and thus antitumor immune response [46]. At later stages, other DAMPs such as HMGB1 are released from dying cancer cells and secreted from activated infiltrated immune cells [13,43,47-49]. Kroemer, Zitvogel and others believed that ICD constitutes a prominent pathway for the activation of the immune system against cancer, which in turn determines the long-term success of anticancer therapies [43,48,50]. The immunogenic characteristics of ICD are mainly mediated by DAMPs that include ecto-CRT, secreted ATP and released HMGB1. Thus, the revised concept ICD would include not only immunogenic apoptosis, but also necrosis, pyroptosis, and autophagic cell death [29,30,42,43,46,51-53].

**Table 1:** Types of cell death and their immunological consequence

**Type of cell death**	**Immunogenicity**
**Apoptosis **(type 1 cell death). This is accompanied by a rounding up of the cell, retraction of pseudopods, reduction of cellular volume, chromatin condensation, nuclear fragmentation, few or no ultrastructural modifications of cytoplasmic organelles, and plasma membrane blebbing, but the integrity of the cell is maintained until the final stages of the process.	**Some forms of apoptosis are non-immunologic, while others are immunogenic.** The pre-apoptotic surface exposure of CRT and HSP70/HSP90 may have a profound impact on the immune response. In addition, the release of HMGB1 during late apoptosis promotes antigen processing by DCs and hence contributes to cytotoxic T-cell activation.
**Autophagic cell death** (ACD; type 2 cell death). Occurs without chromatin condensation but is accompanied by massive autophagic vacuolization of the cytoplasm. The term ACD simply describes cell death with autophagy.	**High.** It may release DAMPs (HMGB1, ATP, and others) and elicit substantial inflammation.
**Necrosis** (type 3 cell death). Characterized by a gain in cell volume, swelling of organelles and rupture of plasma membrane, and subsequent loss of intracellular contents, including HMGB1, ATP, etc.	**High.** This causes release of DAMPs and elicits substantial inflammation and affects local environment.
**Pyroptosis** (or caspase 1-dependent cell death). It is a highly inflammatory form of cell death mediated by the inflammasome and caspase-1 activation, and triggered by various pathological stimuli, such as microbial infection, or stroke, heart attack and cancer.	**High.** It is a highly inflammatory form of cell death due to cytokine release and escape of cytoplasmic contents (DAMPs). However, some pathogens encode immunosuppressive proteins.
**Secondary necrosis.** This is the dissolution of the cell following apoptosis. Some remaining cellular contents are released.	**High.** It is quite immunogenic due to necrosis occurring in apoptotic cells at the late stage.

**Figure 1:**
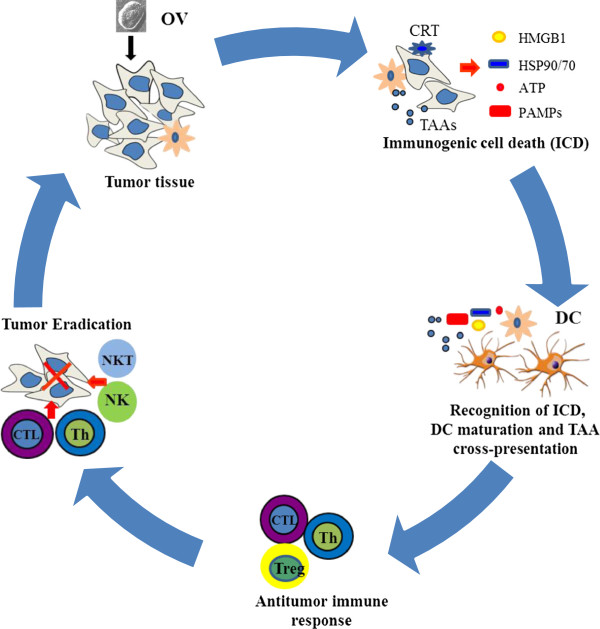
**ICD of cancer cells induced by OVs leads to antitumor immunity.** An OV, delivered either intratumorally or systemically, reaches to tumor tissue and selectively replicates in tumor or/and stromal cells. This leads to induction of death of these cells, presenting “eat me” signals on the cell surface and later release of danger signals from necrotic cells. Apoptotic bodies are engulfed by APC, and TAAs are processed and presented along with MHC complex and costimulatory molecules. The released DAMPs (and PAMPs) activate and mature DCs, and TAAs are cross-presented to naive T cells. This process can be further enhanced at different steps by other immunomodulatory agents (in a combination strategy). The resulting cytotoxic immune response against tumor and associated stromal cells, involving CD4^+^ and CD8^+^ T cells, may help in complete eradication of tumor mass. Additional immunotherapies targeting DCs, T cells, and the immunosuppressive TME can further enhance this antitumor immune response.

Cancer cell death induced by OVs is mostly immunogenic (Table 2). For example, an oncolytic hTERT-Ad induced autophagic cell death in tumor cells and in subcutaneous gliomas, which is immunogenic [54]. Measles virus causes ICD in human melanoma cells [55]. Interestingly, a significant portion of the *in vivo* tumor killing activity by OVs, e.g., vesicular stomatitis virus (VSV) and vaccinia virus (VV), is caused by indirect killing of uninfected tumor cells [4]. OVs also target endothelial cells and tumor vasculature, leading to infection and lysis of endothelial cells, and more necrotic death of cancer cell cells due to disruption of tumor vasculature [4-6,56,57]. As for the release of DAMPs from dying cancer cells, we first reported that cancer cells infected by an oncolytic virus, led to necrotic/apoptotic death pathways and HMGB1 was released into the extracellular milieu [58]. As it turns out, HMGB1 release is a universal phenomenon for OVs, as shown in cancer cells infected with an Ad [59], a measles virus [55], an HSV-2 [60], and a coxsackievirus B3 [61]. Extracellular ATP is another potent danger signal released from OV-infected cancer cells [59,61,62]. Together, tumor cell death and ATP release may prime DC and lead to efficient antitumor immunity [63]. Finally, activated innate immune cells and elicited adaptive anti-cancer immunity as well as inflammatory cytokines kill additional cancer cells and stromal cells, leading to release of DAMPs such as HMGB1 [64]. In summary, these studies strengthen the notion that OVs induce immunogenic types of cell death and present/release a number of danger signals, and TAAs to DCs and immune system to elicit antitumor immune responses (Figure 1 and Table 2).

**Table 2:** OVs induce ICD and/or promote antitumor immunity in animal models or human patients (*)

**Virus**	**Modifications**	**ICD and DAMPs (in vitro)**	**Antitumor Immunity (in vivo)**	**Reference**
**Ad**				
hTERT-Ad	E1a gene driven by hTERT promoter	Immunogenic apoptosis	hTERT-Ad and bortezmib (proteasome inhibition) leads to potent antitumor immunity	[65]
Ad5/3-D24-GMCSF	Ad3 fiber E1a-deleted (RB-selective) GM-CSF +	Enhanced autophagy; ecto- CRT; released ATP and HMGB1	Tumor-specific T cell responses and antitumor efficacy in some patients [clinical trial]	[62]
**HSV**				
G207	R34.5-; ICP6-	NA	Systemic antitumor immunity (CD8^+^ T cells)	[66]
HSV-1716	ICP 34.5 gene mutant	Induction of IFN-γ, CXCL9 and CXCL10	Intratumoral injection increased NK and CD8^+^ T cells	[67]
T-VEC	ICP47-γ34.5 - GM-CSF +	Necrosis/apoptosis (*in vivo*)	Antigen-specific T cell responses and decreases in Treg, Ts, and MDSC in human melanoma patients [clinical trials]	[68,69]
HSV-2 ΔPK mutant	ICP10 PK domain deleted	Apoptosis/Pyroptosis	Dominant induction of CD4^+^ Th1 cells	[70]
**Poxvirus**				
vSP	Spi-1/spi-2-	Necrosis/apoptosis HMGB1 release	NA	[58]
vvDD	tk-/vgf-	Necrosis/HMGB1 and ATP release	CD11b + cells and CD11b^+^Ly6G^+^ cells (DCs and Neutrophils)	[71]
Pexa-Vec	tk-GM-CSF+	NA	Antiviral CTL and antibodies against TAAs in Human HCC patients [clinical trial]	[72]
**Arbovirus**				
VSV-GFP (Indiana serotype)	Marker gene GFP	Induction of IL-28 by virally activated innate immune cells in the TME	IL-28 sensitize cancer cells to NK cell recognition and killing	[73]
VSVgm-icv oncolytic vaccine plateform	Deletion in The M protein at position 51; VSV-GM-CSF+	NA	The antitumor immunity is robust enough to control established tumor. Tumor is infiltrated by a large number of IFNγ-producing T and NK cells	[74]
**Paramyxovirus**				
MV-eGFP (Edmonston strain)	Marker gene EGFP	Released inflammatory cytokines and chemokines; IL-6 and HMGB1	Enhance innate antitumor and melanoma-specific adaptive immunity (*in vitro*)	[55]
MV vaccine-infected tumor cells	Marker gene EGFP	ICD; apoptotic cells phagocytosed by DCs	Allowing DC to mature, produce high level of IFN-α, and cross-present TAAs and production of tumor-specific CD8 T cells	[75,76]

*Notes:

(1). data for T-VEC and Pexa-Vec are from human patients;

(2). NA, not assessed.

Autophagy plays roles in both innate and adaptive immunity [13,77,78], and it is a tightly regulated mechanism that mediates sequestration, degradation, and recycling of cellular proteins, organelles, and pathogens. OVs such as Ad [54,79-81], HSV [70,82,83], reovirus [84-86], influenza virus [87], VSV and encephalomyocarditis virus [88] induce autophagy in cancer cells. Autophagy enhances tumor immunogenicity by two mechanisms. First, dying cells with autophagy (autophagic cell death) selectively release DAMPs such as HMGB1 [89], ATP [52,90], and uric acid [91]. Second, autophagy promotes antigen cross-presentation from cancer cells to DCs and then T cells. Autophagy stimulates antigen processing not only for MHC class II, but also for MHC class I pathway [92,93], as shown for endogenous viral antigens during HSV-1 infection [82], and for cross-presentation of TAAs from uninfected cancer cells [94], and influenza A virus-infected tumor cells [95]. Inhibition of autophagy abolished cross-presentation almost completely, whereas induction of autophagy dramatically enhanced the cross-presentation of TAAs. Interestingly, purified autophagosomes could function as efficient antigen carriers for cross-presentation. These studies demonstrated that autophagy within the antigen donor cells facilitates antigen cross-priming to generate TAA-specific or virus-specific CD8^+^ T cells [75,94,96], which could be further explored as a new strategy to enhance OV-mediated antitumor effects in the future [97].

In summary, ICD and autophagy triggered by a number of OVs provide a highly favorable backdrop for the immune system to respond and generate a potent adaptive antitumor immunity (Table 2).

### Oncolytic viruses as therapeutic cancer vaccines

OVs have been explored as therapeutic cancer vaccines for quite a few decades. Pioneering work done by Lindenmann and Klein in 1967 demonstrated that viral oncolysis of tumor cells by influenza virus increases immunogenicity of tumor cell antigens [98]. A few decades later, Martuza, Toda and others demonstrated that a genetically engineered oncolytic HSV G207 functions as an *in situ* cancer vaccine for induction of specific anti-tumor immunity in CT26 colon cancer model [66]. When this virus is armed with IL-12, the virally expressed IL-12 can work with the OV synergistically to elicit local and systemic anti-tumor immunity [99].

In order to make OVs better therapeutic cancer vaccines, investigators have recently come up with a number of genetically engineered and armed OVs and combination strategies with other anti-cancer agents that may work either additively or synergistically to produce potent oncolysis and antitumor immunity. A number of studies lead us to note two interesting findings. One is that adaptive antiviral immunity may not be all bad. In fact, adaptive antiviral immunity contributes to oncolytic virotherapy by an oncolytic HSV [100], even though it may not be the case for all OVs. The second is that selectivity of oncolytic viral replication may reduce antiviral immunity and toxicity, but it does not improve antitumor immunity [101]. The therapeutic efficacy of an OV is a delicate balance of forces, between effective viral replication and oncolysis, viral clearance by antiviral immunity, and antitumor immunity and factors promoting tumor growth [102,103]. Thus, any combination with immunotherapy should take antiviral immunity into account.

A. Genetic modifications of OVs for enhanced immune responses

Genetic modifications of OVs aim to relieve the inhibition of immune responses by the OVs with deletion of viral immune evasion genes, and to enhance antitumor immune responses by inserting immune-enhancing transgenes into the OV vectors. Clearly, no-armed OVs can elicit antitumor immunity in certain tumor models, as demonstrated with HSV-1 G207, H-1716, MV-EGFP and Coxackievirus B3 [55,61,66,67,104]. However, many studies have shown that immunological effects can vary depending on a number of factors including tumor immunogenicity, stage of the tumor and specifics of the particular OV used. To gain better immunological effects, a number of steps in immune response and multiple cell types can be targeted by armed OVs or by combination strategies. We will discuss some recent studies to illustrate these points.

(1)  Modulating the innate immunity

Toll-like receptors (TLRs) are a family of pattern recognition receptors that recognize PAMPs and DAMPs, and trigger the activation and maturation of DCs. As an example, TLR9 responds to viral dsDNA by recognizing unmethylated CpG sequences; thus CpG rich oligodeoxynucleotides have been used as vaccine adjuvants. Along the same logic, oncolytic DNA viruses enriched with CpG motifs are believed to be stronger immunogens. Raykov et al. have tested this idea in a rat lung hepatoma metastasis model by using autologous tumor cells that were infected with CpG enriched parvovirus and then irradiated. They showed a significant reduction in metastatic rate compared with controls [105]. Cerullo et al. have also tested the anti-tumor effects of an oncolytic Ad enriched with CpG motifs (Ad5D24-CpG) in cancer models [106]. In a syngeneic mouse model with B16-OVA melanoma, Ad5D24-CpG significantly improved tumor control, associated a significant increase in tumor and spleen anti-OVA specific T-cells and a decrease in both number and activation of MDSCs in the tumor.

(2)  Enhancing the cross-presentation and priming of TAAs

Heat shock proteins (HSPs) are a family of proteins that act as molecular chaperones and can be induced or released during cellular stress or necrosis. Once they are exposed on the cell surface or released, they become active DAMPs. Due to their mechanistic abilities to catalyze the folding of proteins and their intracellular translocation, HSPs can bind potential antigens at a necrotic scene and deliver them to a variety of antigen presenting cells [107]. Oncolytic Ads expressing several HSPs, including HSP70, HSP90 and HSF1, a heat shock transcription factor, have been constructed and investigated in tumor models. Indeed, they can function as oncolytic cancer vaccines and can induce an MHC restricted tumor antigen-specific CD8+ T cell response in syngeneic melanoma, colorectal and prostate cancer models in immunocompetent mice [108,109]. In fact, an HSP70-overexpressing oncolytic Ad has been tested in a phase I clinical trial [110].

As we have discussed earlier, autophagy induced in cancer cells has been shown to promote cross-presentation of TAAs.

(3)  Viruses engineered to express cytokines, chemokines and co-stimulatory molecules

Many OVs expressing cytokines (such as IL-2, IL-12, IL-18); chemokines (such as CCL5), or costimulatory molecules (such as B7.1 and CD40L) have been studied and some exciting antitumor immunity and therapeutic results have been documented in animal models and in human cancer patients. Due to space limit, we will focus on the GM-CSF armed OVs in this section.

Viruses have evolved with genes to suppress the immune system in order to survive and gain maximum replication in the hosts [111]. In the context of OVs, they may play yin-yang roles. On one hand, they may increase viral persistence in the tumor leading to better oncolysis; while on the other hand, they may inhibit the immune response to both the virus and cancer, and thus reduce the potency of antitumor immunity. The balancing act between the two is not only a science, but also an art [102,112].

Talimogene laherparevec (T-VEC; formerly JS1/ICP34.5-/ICP47-/GM-CSF or OncoVex), represents a good development to realize the potential as an oncolytic vaccine [113]. First, the authors started to build oHSV-1 from a more potent oncolytic strain JS1 instead of a regular laboratory strain. Then the authors made a number of mutations of viral genes based on previous findings. Deletion of the ICP34.5 gene would result in enhanced tumor cell killing. Mutation in ICP47 serves two functions. One is to increase the expression of the HSV US11 gene, which enhances replication of HSV ICP34.5 mutants in tumors. As ICP47 also functions to block antigen processing in HSV infected cells, this mutation was also anticipated to improve the immune stimulating properties of the virus. Finally, in order to provide viruses with maximum immune stimulating properties, the human GM-CSF-encoding gene was inserted into the JS1/34.5-/47- backbone. The data collected at the time indicated that the resulting virus T-VEC acts as a powerful oncolytic agent. The continued work in multiple clinical trials confirmed and extended the original findings.

Genetically engineered vaccinia virus (VV) is another good example. The deletion of viral genes encoding thymidine kinase (*tk*) and vaccinia growth factor (*vgf*) makes it a highly tumor-selective one, called vvDD [114]. These mutations restrict virus replication to cells that overexpress E2F (positively regulate cellular TK expression) and have constitutively activated epithelial growth factor receptor pathway. When it is armed with GM-CSF gene, its antitumor immunity and cytotoxicity were further enhanced [115].

GM-CSF mediates antitumor effects by recruiting NK cells and by induction of tumor antigen-specific cytotoxic T cells through the action of APCs. Some of most promising OVs are Ad, HSV or VV armed with the human GM-CSF gene. All three have been tested in multiple clinical trials. One of the Ad versions, Ad5-D24-GMCSF, induces antitumor immunity in cancer patients. Of the 16 patients evaluable, two had complete response, and 5 stable disease [116]. Another version, a serotype 5/3 chimeric Ad expressing GM-CSF, has achieved similar immune and clinical responses in cancer patients [117].

The HSV version is T-VEC. In animal models, this virus acts as a powerful agent with enhanced oncolytic, immune stimulating, and anti-tumor properties [113]. In a phase I trial, the virus was generally well tolerated. Virus replication, local reactions, GM-CSF expression, and HSV antigen-associated tumor necrosis were observed. After treatment, most patient biopsies contained residual tumor of which 14 showed tumor necrosis or apoptosis [68]. In a phase II trial, patients’ unresectable metastatic melanomas were treated with multiple intratumoral injections of the virus, then clinical responses, survival and safety were monitored. The overall response rate by RECIST was 26%, with complete response in 8 out of 50 patients [118]. Direct injection of this virus induced local and systemic antigen-specific T cell responses and decreased CD4^+^FoxP3^+^ regulatory T cells (Treg), CD8^+^FoxP3^+^ suppressor T cells, and myeloid-derived suppressive cells (MDSC) in patients exhibiting therapeutic responses [69]. T-VEC has an approximately 30% response rate against systemic disease, following local injection into accessible tumors. A pivotal phase III trial for T-VEC has just been completed in melanoma, and a phase III trial in head and neck cancer is also underway [119].

The main findings of the phase III trial were presented orally at the 2013 American Society of Clinical Oncology Annual Meeting (Abstract no. LBA9008) [120]. In the OPTiM trial, 436 patients with unresectable stage IIIB-IV melanoma were randomized 2:1 to receive either T-VEC injected into the lesions directly or by ultrasound guidance, or GM-CSF administered subcutaneously. There were 295 patients in the T-VEC group and 141 participants in the GM-CSF arm. The overall durable response rate (DRR) was 16.3% for patients who took T-VEC, compared with 2.1% among participants who received just GM-CSF. The objective overall response (ORR) rate was 26.4% among the T-VEC group, including 10.8% with a complete response, compared with an ORR of 5.7% and a complete response of 0.7% in the GM-CSF group. This is the first phase III trial demonstrating the efficacy of an OV immunotherapy.

Pexa-Vec (pexastimogene devacirepvec; JX-594; TG6006) is an oncolytic poxvirus armed with the GM-CSF gene and it has undergone multiple phase I/II clinical trials and obtained exciting clinical responses in liver cancer patients [5,72]. Viral replication and expression of GM-CSF and induction of antitumor immunity were all detected. Interestingly, survival duration of patients was significantly related to viral dosage, with median survival of 14.1 months compared to 6.7 months on the high and low dose, respectively [72]. In a related study, Pexa-Vec has been shown to induce antibody-mediated complement-dependent cancer cell lysis in humans. The authors have identified about a dozen of TAAs using serological expression cloning approach [121].

B. Combination with other immunotherapy regimens

As a form of immunotherapy [7-9], OVs in combination with other immunotherapy regimens would make sense if they function additively or synergistically to exert potent and sustained antitumor immunity. Investigators have combined OVs with DC-mediated active immunization, adoptive T cell transfer, or other immune-modulators to regulate other immune components in order to generate potent antitumor immunity and improve overall therapeutic efficacy.

OVs and DC-mediated cancer vaccines can be combined to improve the efficacy. A recent study has showed that intratumoral OV-induced inflammation is a precondition for effective antitumor DC vaccination in mice [122]. This regimen combining tumor-targeted DC vaccine with ongoing OV-induced tumor inflammation elicited potent antitumoral CD8^+^ T cell responses and marked tumor regression and successful eradication of pre-established lung colonies, a model for tumor metastases. One unexpected finding has been that depletion of Tregs abrogated antitumor cytotoxicity. As such, Tregs are essential for the therapeutic success of multimodal and temporally fine-adjusted vaccination strategies. These results highlight tumor-targeting OVs as attractive tools for eliciting effective antitumor responses upon DC vaccination [122].

CD8^+^ T cells are critical for the efficacy of VSV virotherapy, and yet these cytotoxic T cells are induced against both virally encoded and tumor-associated immunodominant epitopes [123]. Vile group and others have tested various immune interventions to increase the frequency/activity of activated antitumoral T cells in the context of OVs. Treg depletion had a negative therapeutic effect because it relieved suppression of the antiviral immune response, leading to early viral clearance. In contrast, increasing the circulating levels of tumor antigen–specific T cells using adoptive T cell transfer therapy, in combination with intratumoral virotherapy, generated significantly improved therapy over either adoptive therapy or virotherapy alone [123]. In addition, incorporation of a TAA within an OV increased the levels of activation of naïve T cells against the antigen, which translated into increased therapeutic efficacy [123-125]. Therefore, these studies have demonstrated that combination strategies that enhance immune activation against TAAs can be integrated to enhance the efficacy of virotherapy [123-125].

A number of studies have utilized a heterogeneous “prime-boost” regimen in oncolytic immunotherapy. VSV engineered to express chicken ovalbumin (OVA) could efficiently treat mice bearing B16 melanomas expressing OVA as a model tumor antigen [123,126]. Mice treated with VSVova developed potent anti-ova immunity and many of their B16-ova tumors completely regressed. In another study, a similar regimen using Semliki Forest virus (SFV) followed by VV, or vice versa, leads to enhanced antitumor effect against a murine ovarian cancer model [127]. Infection with SFV-OVA followed with VV-OVA leads to enhanced antitumor effects through a combination of viral oncolysis and antigen-specific immunity. The more clinically relevant strategy has been to develop OVs that express self-tumor antigens and utilize syngeneic tumor models with self-tumor antigens. This is much more challenging, yet investigators have come up with innovative approaches. One strategy was to use replicating OVs to boost antitumor immunity primed by a nonreplicating Ad-based vaccine [128,129]. Bridle and colleagues took a heterologous “prime boost” approach using non-replicating Ad expressing self-antigen hDCT (Ad-hDCT) as prime intramuscularly, then boosted with replicating VSV-hDCT by intravenous administration in a metastatic B16 melanoma model. The immunological results are very intriguing but consistent with other prime-boost regimens. While VSV-hDCT treatment alone elicited a strong T-cell response towards viral antigens, the prime boost regimen completely polarized the adaptive immune response towards the hDCT tumor antigen. Using such a prime-boost regimen, a large percentage of mice were cured of tumors.

T and NK cells express several members of the TNF receptor (TNFR) family specialized in delivering a costimulatory signal. Engagement of these receptors is typically associated with proliferation, elevated effector functions, resistance to apoptosis, and differentiation into memory cells. Therefore, agonist monoclonal antibodies (mAb) against these molecules have been used to stimulate antitumor T and NK cells in cancer therapy settings [130]. It makes sense to combine an OV with such a mAb for therapeutic purpose. Combining an OV with a potent agonist antibody specific for the costimulatory molecule 4-1BB showed improved therapeutic outcomes [71]. Combination of an OV with an immunomodulatory mAb that blocks T-cell checkpoint blockade receptors such as CTLA4 has also generated promising results [131].

To overcome the heterogeneity nature of tumor, a group of investigators have combined complementary OVs to attack cancers in distinct ways to improve therapeutic outcome [132]. Two genetically distinct viruses, VSV and VV, were used to eliminate the risk of recombination. They found that VV synergistically enhanced VSV antitumor activity, dependent in large part on the activity of the VV *B18R* protein [132]. Recently, another combination of two OVs applied at multiple low doses to tumor models of the Syrian hamster as an immune-competent model enhance antitumor efficacy through the induction of tumor-specific immunity and circumvention or mitigation of antiviral immune responses [133].

In most cases, combinations with other immunotherapy regimens have generated enhanced antitumor immunity and better therapeutic outcomes. However, some of these studies lead to some unexpected conclusions in the context of OVs. First, adaptive antiviral immunity contributes to oncolytic virotherapy in the context of oHSV [100], but high levels of VSV-associated immunogenicity distracted immune response away from priming for tumor-specific T cells [134]. Second, two studies showed that Treg cells are needed for optimal therapeutic results, due to either prevention of early viral clearance or due to the compensatory induction of MDSCs in Treg-depleted and thus vigorously inflamed tumors which prevent oncolysis-assisted DC vaccination [122,123]. Third, in prime-boost strategies using two different OVs, the immunological outcomes depend upon the order of vaccination – Ad followed by VV was not only better than either virus alone but better than VV followed by Ad [133]. This is not too surprising as similar observations have been made previously with classic replication-deficient viral vectors. However, this means that investigators will need to assess their scheduling carefully in all combination regimes with OVs.

C. Modulation of the TME to promote viral replication and antitumor immunity

The TME can be modulated not only to promote OV viral replication and oncolysis, but also the activation, persistence and activities of antitumor immune cells. We will discuss only a few such strategies that have been applied to OV regimens. Innate immune cell recruitment and activation have been shown to be deleterious to the efficacy of OVs [135-138]. As an example, NK cells impede glioblastoma virotherapy through NKp30 and NKp46 natural cytotoxicity receptors [139]. One major trigger for the activation of innate immune cells is the interferon (IFN) response induced by viral infection.

Quite surprisingly, one class of small molecules that inhibit the IFN responses is the inhibitors of histone deacetylases (HDACi) [140,141]. HDACs can influence epigenetic modifications of histones and chromatin, and a number of other cellular regulatory proteins, leading to inhibition of the cellular antiviral response. In one study, the authors showed that two HDACi, MS-275 and vorinostat, markedly enhance the infection and spread of VSV and VV in cancer cells and primary human tumor tissue explants *in vitro*, and in multiple animal models. The authors found that reduced cellular IFN responses and enhanced virus-induced apoptosis may explain the increased viral replication and oncolytic activity [142]. It has been shown that HDACi valproic acid (VPA) augmented antitumor efficacy of oncolytic HSVs [143]. VPA lessens NK cell action against OV-infected glioblastoma cells by inhibition of STAT5/T-BET signaling and generation of IFN-γ [144]. When administered prior to HSV inoculation in an orthotopic glioblastoma mouse model, VPA resulted in a reduced recruitment of NK and macrophages into tumor-bearing brains at early time post-HSV infection. Interestingly, the recruitment of these cells rebound robustly at a later time point. The authors corroborate these findings *in vitro* by demonstrating that VPA reduces NK cell-mediated cytotoxicity and production of gamma interferon. VPA has a profound suppressive effect on human NK cells by inhibiting NK cell cytotoxicity via downregulation of cytotoxic proteins granzyme B and perforin. In addition, suppression of gamma IFN (IFN-γ) production was associated with decreased STAT5 phosphorylation and dampened T-BET expression. These results demonstrate that HSV virotherapy of glioblastoma is limited partially by an antiviral NK cell response, which can be modulated by VPA or other agents to enhance cancer virotherapy [139].

A recent study revealed an unexpected property of HDACi on adaptive immunity [145]. A class I-specific HDAC inhibitor, MS-275, induced lymphopenia which led to selective depletion of bystander lymphocytes and regulatory T cells while allowing expansion of antigen-specific secondary responses. Coadministration of vaccine (oncolytic VSV) with the drug during the boosting phase focuses the immune response on the tumor by suppressing the primary immune response against the vaccine vector and enhancing the secondary response against the tumor antigen. Evidence suggests that MS-275 can orchestrate a complex array of effects that synergize immunotherapy and viral oncolysis. Overall, MS-275 enhanced efficacy, suppressed autoimmunity and thus improved the therapeutic index [145]. In addition, it is tempting to point out that such as HDACi or inhibitors of DNA methylation have been used to enhance the immunogenicity of tumor cells by upregulation of TAAs [146,147] , and HMC class I antigens and antigen presentation machinery [148,149], and thus enhance cancer immunotherapy [146].

The TME is characterized as chronic indolent inflammation in which the effector function of tumor-infiltrating lymphocytes (TILs) is severely impaired. This TME makes the effector cells generated by cancer vaccines malfunctional and impotent. Recent studies have shown that costimulation with TLR ligands may greatly enhance the efficacy of immunotherapy including cancer vaccines [150]. Injection of oncolytic VSV leads to tumor regression in established B16ova melanoma model. This effect is in part due to the induction of innate immunity against the viral infection that is mediated by MyD88- and type III IFN-, but not TLR4-, signaling pathway [151]. Strikingly, intratumoral injection of lipopolysaccharide (LPS), a TLR-4 agonist, leads to activation of different innate immune pathways and significantly enhances the local oncolytic therapy by VSV. This antitumor activity is further enhanced by co-recruiting a potent antitumor, adaptive T-cell response by using a VSV engineered to express ova, the artificial tumor antigen, in combination with LPS [152]. However, this study also highlights unforeseen dangers of combination therapies in which an immunotherapy may systemically sensitize the host (potentially a human patient) to a cytokine shock-like response triggered by systemic delivery of an OV.

**Figure 2:**
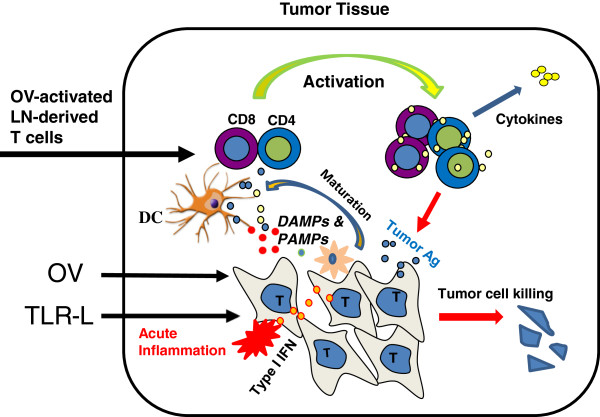
**A model of how TILs in the TME are rescued to exert their effector functions by TLR ligands-reactivated DCs in the TME.** TILs activated by oncolytic virotherapy or other cancer vaccines migrated from lymph nodes to the tumor tissues may require *in situ* activation by tumor–infiltrated DCs. However, the tumor-infiltrated DCs (TIDCs) are immunologically suppressed in the TME, but can be activated by TLR ligands or/and other TLR3/9 ligands (TLR ligands) through type I IFN-dependent signaling. Some OVs themselves or their products (such as dsRNA) can function as TLR ligands. The functionally reactivated TIDCs can acquire, process, and present TAAs to reactivate TILs to exert their functions. This model is modified from Xiao H et al., 2013 [153].

The effector function of CD8^+^ TILs could be rescued by converting the chronic inflammation milieu to acute inflammation within tumors. Injection of TLR3/9 ligands (polyI:C/CpG) into a tumor during the effector phase of lentivector (lv) immunization effectively rescued the function of lv-activated CD8^+^ TILs and decreased the percentage of Treg within the tumor, resulting in a marked improvement in the antitumor efficacy of the immunization [153]. We provided a working mechanism by showing that rescue of the effector function is most likely dependent on production of type-1 IFN in the tumor that can mature and activate tumor-infiltrating DCs. It is worth noting that many OVs or their products can be recognized as PAMPs by TLRs or other pattern recognition receptors (PRRs) expressed by DCs, thus stimulating DCs [154]. For example, oncolytic parvovirus H-1 activates DCs partially through TLR3 and TLR9 [155]. Reovirus can escape the endosomes of DC and viral dsRNA triggers non-TLR3 receptor (other PRR receptor) to induce IFN-γ production, and prime adaptive antitumor immunity [156]. Based on these studies, we have presented a model how TLR ligands rescue the immunological function of the TILs (Figure 2). In this model, type I IFN, produced via TLR-TLR ligand signaling and activation of the gene, plays some key roles in reactivating tumor-infiltrating DCs (TIDCs), which reactivate TILs. Some OVs can function well as ligands for TLRs.

D. **Combination with cyclophosphamide for enhanced antitumor immunity**.

The immune system makes a crucial contribution to the antitumor effects of conventional chemotherapy- and radiotherapy-based cancer regimens [157]. Cyclophosphamide (CPA) is an alkylating agent and a classic chemotherapeutic compound. It induces genotoxic stress, apoptosis and/or cell cycle arrest. Recent studies indicate that it can enhance viral replication of OVs and adaptive antitumor immunity *in vivo*, thus resulting in better efficacy. CPA functions to promote oncolytic virotherapy mainly via two mechanisms. (1). CPA enhances viral replication by suppressing antiviral innate immunity. Chiocca and associates have first applied CPA in combination with oHSV virotherapy based on the rationale that CPA would reduce antiviral immunity and thus augment viral replication to enhance oHSV efficacy [135-137,158]. They discovered that pretreatment with a single dose of CPA could enhance both the level and duration of viral replication of HSV within treated tumors [137,158]. Similar enhancement was also reported for oncolytic Ad [159]. The CPA-enhanced viral replication is well correlated to the significantly enhanced antitumor activities [137,158,160]. Nevertheless, it is worth noting that not all combinations of an OV with CPA will work. In fact, VSV-induced immune suppressor cells generate antagonism between intratumoral OV and CPA [161]. (2). CPA enhances adaptive antitumor immunity induced by OVs. This is most likely through selective depletion and inhibition of Treg cells by low dose of CPA [162-164]. CPA pretreatment followed with virotherapy leads to significantly enhanced antitumor immunity in tumor models of immunocompetent mice, as demonstrated with oncolytic HSV [160], Ad expressing gp96 [165], and VV expressing HPV E7 [166]. In addition, CPA can enhance antitumor activity of adoptively transferred immune cells through the induction of “cytokine storms” [167]. In the last few years, CPA in combination with OV has been tested in human cancer patients. Oncolytic Ad given together with metronomic CP increased cytotoxic T cells and induced Th1 type immunity on a systemic level in most cancer patients tested [62,168]. In summary, CPA has emerged as a clinically feasible agent that can suppress Tregs and allow more effective induction of antitumor responses , in the settings of cancer vaccines and other immunotherapy strategies [169].

## Conclusions

In the capacity of cancer vaccines, OVs exert two of the most important functions: (1). They kill cancer cells and associated stromal cells directly by oncolysis or indirectly by anti-angiogenesis, vascular-targeting and by-stander effect; and (2). They efficiently present/release DAMPs and PAMPs (signal 0) and present TAAs (signal 1) to DCs in order to trigger a TAA-specific antitumor immunity. However, OVs by themselves may not be enough because the immunosuppressive TME often impairs the functions of both innate and adaptive immune cells. Therefore, investigators have designed a number of combination strategies to overcome the TME and potentiate the antitumor immunity initiated by the OVs.

We have discussed a variety of combination strategies with OVs to boost the antitumor immunity and sustain their cytotoxic activity against cancer in the TME. These strategies are targeted at the stages of immunogenicity of (dying) cancer cells, the process of antigen presentation, the potency of immune cells, and the overall immunological status of the TME, the latter of which can be modulated via blockade of immune checkpoints, depletion of immunosuppressive cells, and/or further activation of immune effector cells by either active immunization, or/and by adoptive T cell transfer. We envision that antitumor immunity elicited by OVs properly armed or rationally combined would kill not only residual cancer stem cells and “differentiated” cancer cells in primary cancer and metastases, but also maintain micrometastases in dormant status. This is a key for treating metastatic cancer.

In phases I-II clinical trials, several OVs armed with either GM-CSF or CD40L showed specific antitumor immunity, significant antitumor activity and clinical responses in a significant fraction of cancer patients. T-VEC has demonstrated efficacy in a phase III trial for melanoma patients while Pexa-Vec has been tested in a phase IIb trial for patients with hepatocellular carcinoma. It is likely that one or both of them may be approved by FDA in the near future. Looking forward, this new class of therapeutic cancer vaccines is promising and more efforts should be invested in both preclinical and clinical investigations.

## Abbreviations

OV: Oncolytic virus; DCs: Dendritic cells; HDAC: Histone deacetylase; HDACi: Inhibitor of HDAC; TAAs: Tumor-associated antigens; DAMPs: Damage-associated molecular pattern molecules; PAMPs: Pathogen-associated molecular pattern molecules; TME: Tumor microenvironment; PKR: The double stranded RNA (dsRNA)-activated protein kinase; ICD: Immunogenic cell death; NICD: Non-immunogenic cell death; CRT: Calreticulin; Ecto-CRT: Surface-exposed CRT; TILs: Tumor-infiltrated lymphocytes; MDSCs: Myeloid-derived suppressive cells; GM-CSF: Granulocyte-macrophage colony-stimulating factor; LPS: Lipopolysaccharide; CPA: Cyclophosphamide; OVA: Chicken ovalbumin; VPA: Valproic acid; Ad: Adenovirus; HSV: Herpes simplex virus; MV: Measles virus; HPV: Human papilloma virus; SFV: Semliki Forest virus; VV: Vaccinia virus; VSV: Vesicular stomatitis virus; HMGB1: High mobility group box 1; RECIST: Response Evaluation Criteria In Solid Tumors; IFN: Interferon; mAb: Monoclonal antibody; TIDCs: Tumor-infiltrated dendritic cells; TLR: Toll-like receptor; HSPs: Heat-shock proteins; T-VEC: Talimogene laherparevec; Pexa-Vec: Pexastimogene devacirepvec.

## Competing interests

DLB serves as a scientific advisor for and has financial interest with Jennerex Biotherapeutics, a biotech company developing oncolytic viruses. The other authors declare no conflict of interest.

## Authors’ contributions

ZSG collected and read relevant papers; designed and drafted the manuscript. YH proposed the original hypothesis presented in Figure 2. All other authors have made suggestions to the manuscript. All authors have read and approved the final manuscript.
